# Reconstitution of lamin assembly on nuclear pore complex-containing membranes

**DOI:** 10.1101/2025.07.28.667287

**Published:** 2025-07-30

**Authors:** Ross TA Pedersen, Yinyin Zhuang, Andres V Reyes, Shou-Ling Xu, Xiaoyu Shi, Yixian Zheng

**Affiliations:** 1 Department of Embryology, Carnegie Institution for Science; Baltimore, MD.; 2 Department of Developmental and Cell Biology, University of California, Irvine; Irvine, CA.; 3 Department of Plant Biology, Carnegie Institution for Science; Stanford, CA.; 4 Carnegie Mass Spectrometry Facility, Carnegie Institution for Science; Stanford, CA.; 5 Department of Chemistry, University of California, Irvine; Irvine, CA.; 6 Department of Biomedical Engineering, University of California, Irvine; Irvine, CA.

## Abstract

Intermediate filaments called lamins line the metazoan nuclear envelope and organize the nucleus and genome. Unlike actin and microtubules, purified intermediate filament proteins assemble into non-physiological structures, making it difficult to connect lamin functions to their assembly and regulation. To overcome this challenge, we reconstituted lamin assembly without nuclear assembly using interphase *Xenopus laevis* egg extracts that recapitulate physiological context. Mimicking nucleoplasm conditions triggers dispersed lamin assembly in egg extracts. Such ectopic lamin assembly occurs on nuclear pore complex-containing membranes, but does not recruit known nuclear lamina components, demonstrating that lamin assembly is separable from the rest of the nuclear lamina and nucleus. This assembly assay in the physiological context of cellular components opens the door to mechanistically dissecting nuclear lamina function in nuclear organization.

## Introduction

The nuclear lamina is a thin protein layer on the inner surface of the nuclear envelope in metazoan cells. It is important for shaping the nucleus, but it also interacts with and organizes components of the nuclear envelope (1–3) and controls gene expression and genome structure (4–6). The major structural component of the nuclear lamina is a meshwork of intermediate filament polymers composed of protein subunits called lamins. We know surprisingly little about how the basic biochemical properties of lamins give rise to their myriad roles in the nuclear lamina. Why lamin assembles nearly exclusively under the inner nuclear envelope and whether proteins of the inner nuclear envelope are required for proper lamin assembly or organization are examples of fundamental questions that remain unanswered.

Unlike actin and microtubules, which polymerize into structurally similar filaments either in pure form or in cells (7, 8), proper assembly of intermediate filaments – including lamins – requires cellular context. Intermediate filaments assembled *in vitro* from purified subunits are structurally polymorphic (9–11). Lamins behave particularly poorly *in vitro*. Most isoforms transiently assemble into filaments before aggregating laterally at steady state into non-physiological structures called paracrystals (12–15). Structural studies of both cytoplasmic intermediate filaments and lamins *in situ* paint a more consistent picture, revealing that ~10 nm-diameter vimentin filaments comprise five protofibrils (16), while lamins are composed of a single protofilament ~4 nm in diameter (17, 18). Mutational analysis also emphasizes the shortcomings of studying assembly of purified lamins; mutations that cause aberrant lamin assembly in nuclei do not necessarily cause corresponding *in vitro* assembly defects for purified lamins (19). Thus, for studies of lamin assembly to be fruitful, it is critical to develop an assembly assay that recapitulates the physiological conditions for lamin polymerization found in the nucleus.

*Xenopus laevis* egg extracts contain assembly-competent lamin-B3 and have been invaluable for understanding physiological regulation of other cytoskeletal assembly mechanisms (20) and the cell cycle (21) without the constraint of a cell boundary. Chromatin added to interphase-arrested *Xenopus* egg extracts assembles into nuclei with functional nuclear pore complexes (NPCs) and a lamin meshwork composed primarily of lamin-B3, an egg- and embryo-specific isoform (22). Reconstitution using simpler substrates, including chromatin- and Ran-coated beads, has helped identify key biochemical signals that orchestrate nuclear assembly (23, 24). Unfortunately, these approaches have been less successful in studying nuclear lamina and lamin filament assembly because it is difficult to separate these processes from nuclear assembly. Using these existing egg extract reconstitution approaches, it impossible to distinguish whether an assembly mechanism of interest pertains directly to lamin assembly, or indirectly through affecting nuclear assembly.

As it stands, we are poorly equipped to understand basic lamin cell biology, let alone how lamins organize the nucleus and genome. It is increasingly clear that the nuclear lamina impacts differentiation through controlling genome organization (6) and the transcriptional impact of chromatin states (25). Knowing the minimal conditions and components necessary for physiological lamin assembly would help us to biochemically dissect how the entire nuclear envelope region, complete with heterochromatin, is assembled to carry out these functions.

## Results and Discussion

We reasoned that we might be able to find conditions that support lamin assembly without nucleus assembly in *Xenopus* egg extracts. We mimicked the conditions of nucleoplasm in crude egg extracts by adding Ran-L43E, a mutant that behaves as Ran-GTP, or the same buffer without Ran-L43E as a control. In interphase cells, Ran-GTP is concentrated in the nucleoplasm where it releases nuclear import receptors from their cargos. It also contributes to nuclear assembly (24, 26) by supporting NPC formation through releasing NPC components (nucleoporins) from nuclear import receptors (27, 28). Nuclear import receptors also inhibit lamin-B3 assembly (29), leading us to believe that releasing them from their cargos with Ran L43E may stimulate assembly. Epifluorescence imaging of samples stained by immunofluorescence revealed lamin-B3 filaments in Ran-L43E-supplemented crude extracts (Fig. S1A). However, crude extracts are not optimal for systematically investigating lamin assembly. Lamin-B3 no longer assembles in them if they are frozen and thawed and they contain heavy components that make them unsuitable for analysis by sucrose gradient sedimentation (see below). They also contain pigment granules that exploded during Stimulated Emission Depletion (STED) microscopy, precluding super-resolution imaging (Fig. S1B).

**Figure 1:**
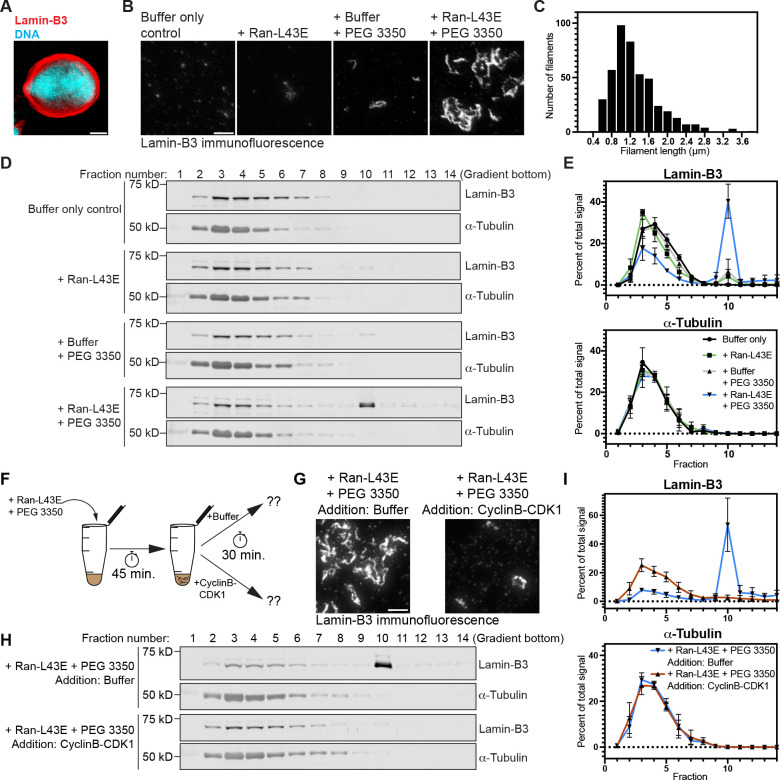
Reconstitution of regulated lamin-B3 assembly in the absence of nuclear assembly **(A)** Two-color confocal slice of a nucleus assembled around demembranated sperm chromatin in “cleared” *X. laevis* egg extracts. Lamin-B3 in red, DNA in cyan. Scale bar: 5 μm. **(B)** Maximum intensity projections from z-stacks of STED microscopy lamin-B3 immunofluorescence of cleared egg extracts treated as indicated. Ran-L43E was added to ~25 μM. Scale bar: 2 μm. **(C)** Lamin-B3 filament length distribution measured in extracts treated with Ran-L43E and PEG 3350 as shown in the rightmost image of panel B. Filament lengths from two biological replicate experiments were lognormally distributed, both as individual replicates and as a combined dataset, as displayed (D’Agostino and Pearson test: K^2^ = 4.35, p = 0.1136, n = 436). **(D)** Example immunoblots showing how lamin-B3 and α-tubulin (as a control) sediment on sucrose gradients for cleared egg extracts treated, as indicated, with the same conditions as in panel B. **(E)** Quantification of the fraction of total lamin-B3 and α-tubulin present in each fraction of sucrose gradients from three biological replicates of the experiment depicted in panel D. Error bars are standard deviation (SD). **(F)** Schematic of an experiment designed to test whether lamin-B3 assembly in our reconstitution assay is reversed by CyclinB-CDK1 function. **(G)** Maximum intensity-projected STED lamin-B3 immunofluorescence images of the experiment schematized in panel F. Scale bar: 2 μm. **(H)** Sucrose gradient sedimentation was used to assay lamin-B3 assembly state in the experiment schematized in panel F. Example immunoblots are shown. **(I)** Quantification (as in panel E) of sucrose gradients from three biological replicates of the experiment in panels F and H. Error bars: SD.

To overcome these challenges, we clarified crude egg extracts by centrifugation to create a partially clarified cytoplasm that contains a subset of cellular membranes (30). These “cleared” extracts retain the ability to form nuclei around added sperm chromatin even after being stored frozen (Fig. 1A). STED imaging revealed that Ran-L43E alone hardly triggered any lamin-B3 assembly in cleared egg extracts (Fig. 1B) as compared to what we qualitatively observed in crude egg extracts (Fig. S1A). A major component of crude egg extracts that is removed while making cleared egg extracts is glycogen (30), which may function as a crowding agent that recapitulates the elevated colloid osmotic pressure of nucleoplasm (31, 32). We added an inert crowding agent (here, PEG 3350) to mimic the colloid osmotic pressure of nucleoplasm and found that under this condition, Ran-L43E stimulated considerable lamin-B3 assembly as visualized by STED microscopy (Fig. 1B). Lamin-B3 filaments tended to overlap with each other in a meshwork-like manner, but when we measured isolated individual filaments, they had lengths that were lognormally distributed with a median of 0.937 μm (Fig. 1C). The length distribution of lamin filaments in nuclei as revealed by cryo-electron tomography is similarly right-skewed (17) with some variability between cell types (33).

We next used sucrose gradient sedimentation as a semi-quantitative assay of lamin-B3 assembly in cleared egg extracts. Extract samples treated with control or lamin-B3 assembly conditions were separated down linear 5–50% sucrose gradients, then fractions from the gradient were analyzed by immunoblotting (Fig. 1D–E). Conditions that give rise to lamin-B3 assembly visible by immunofluorescence (Fig. 1B) also cause lamin-B3 to sediment into the bottom half of the gradient, primarily to the tenth fraction (Fig. 1D–E).

To determine whether our assembled lamin-B3 filaments are regulated by the master mitotic kinase known to disassemble both the nucleus and nuclear lamina, CyclinB-CDK1, we assembled lamin-B3 filaments in egg extracts, then added either CyclinB-CDK1 or buffer as a control and incubated for an additional 30 minutes (Fig. 1F). Lamin-B3 structures incubated with buffer remained detectable by immunofluorescence and sucrose gradient sedimentation after 30 minutes, while treatment with CyclinB-CDK1 for the same period resulted in complete lamin-B3 disassembly (Fig. 1G–I), as expected based on the role of CyclinB-CDK1 in triggering disassembly of lamin filaments in mitosis (34, 35). Thus lamin-B3 filaments assembled in our nucleus free conditions are properly regulated by this major cell cycle kinase.

In addition to being a useful readout of lamin-B3 assembly, our sucrose gradients also partially purify lamin-B3 filaments assembled in our assay from other extract components, which allowed us to identify proteins associated with assembled lamin-B3 using mass spectrometry. Since lamin-B3 filaments reproducibly sediment primarily to the tenth fraction in our 5–50% sucrose gradient experiments (Fig. 1D–E, Fig. 1 H–I), we compared proteins identified in the tenth sucrose gradient fraction for extracts treated with lamin-B3 assembly conditions (1% PEG 3350, 25 μM Ran-L43E) to those identified in the same fraction for control extracts treated with buffer only, which triggers no lamin-B3 assembly as detected by immunofluorescence or sucrose gradient sedimentation (Fig. 1B, Fig. 1D–E).

**Figure 2:**
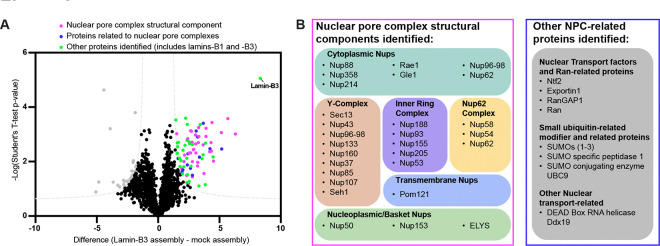
Proteomic analysis highlights the intimate relationship between lamin assembly and nuclear pore complexes **(A)** Volcano plot of enrichment of proteins identified by mass spectrometry in sucrose gradient fractions that contain lamin-B3 filaments compared to proteins identified in the same fraction under mock assembly conditions as determined by two-sided T-test. Gray dashed lines are nonlinear significance thresholds (False Discovery Rate = 5%, S_0_ = 1). Gray datapoints on the left are proteins significantly enriched in mock assembly samples. Datapoints on the right are 65 unique proteins significantly enriched with assembled lamin-B3. 26 of them were nucleoporins (magenta). 10 of them were associated with the NPC or related to nuclear transport (blue). Of the remaining 29 (green), 3 were lamins, including the most significant and abundant protein identified, lamin-B3 (labeled). **(B)** Left: all but 4 NPC structural proteins were found associated with lamin-B3 structures, including components from every subcomplex. Right: examples of NPC- and nuclear transport-related proteins identified associated with lamin-B3 structures.

We found 65 unique proteins significantly associated with lamin-B3 filaments in our reconstitution assay (Fig. 2A). As expected, the most significantly enriched protein identified was lamin-B3, and lamin-B1 was also significantly enriched (Fig. 2A, Table S1). Of the remaining unique proteins enriched in the lamin-B3 filament fraction, 26 are nucleoporins, representing nearly every NPC component (Fig. 2B). The only nucleoporins not present in the fraction that contained lamin-B3 filaments were the cytoplasmic nucleoporin CG1 and the nucleoplasmic nucleoporin TPR, each peripheral nucleoporins that are known to associate with the NPC less stably than core nucleoporins (36). The transmembrane nucleoporins NDC1 and GP210 were also absent, although we did identify the transmembrane nucleoporin Pom121. Another 10 of the identified proteins are related to nuclear transport or associated with NPCs (Fig. 2C). Surprisingly, no canonical nuclear lamina proteins (e.g., Lamin-B Receptor, LEM domain-containing proteins) were found associated with lamin-B3 filaments (Table S1), despite being components of the nuclear lamina in nuclei assembled in *Xenopus* egg extracts (37–39).

Lamin filaments assemble on the inner surface of the nuclear envelope and are non-randomly distributed with respect to NPCs. NPCs tend to cluster within 100 nm of lamin filaments, although the two structures do not tend to colocalize (40). Our identification of many nucleoporins in sucrose gradient fractions containing lamin-B3 filaments suggests that these filaments assemble on membranes containing NPCs that resemble the inner nuclear envelope. Indeed, membrane structures laden with NPCs called annulate lamellae have been observed in *Xenopus* egg extracts (41, 42). Annulate lamellae are stacks of endoplasmic reticulum-derived membranes that store partially assembled NPCs to be inserted into the nuclear envelope (43).

**Figure 3:**
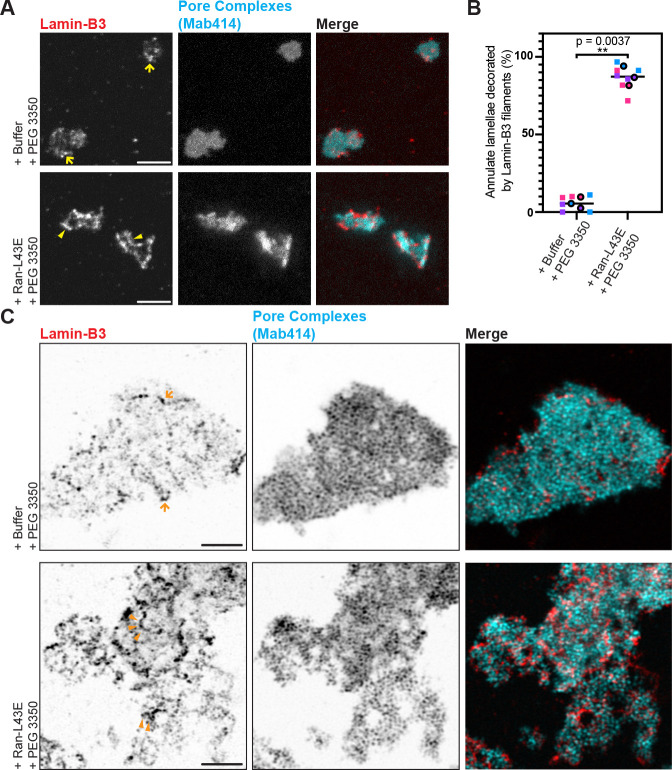
Lamin-B3 assembly occurs on annulate lamellae in the absence of nuclei **(A)** Maximum intensity-projected two-color STED immunofluorescence images of lamin-B3 (red) and pore complexes (cyan, labeled by Mab414), in cleared egg extracts supplemented as indicated. Arrows in the Buffer/PEG 3350 image highlight punctate lamin-B3 signal, while arrowheads in the Ran-L43E/PEG 3350 image point to examples of filaments. Scale bars: 2 μm. **(B)** Quantification of the experiment from panel A: percentages of annulate lamellae decorated by lamin-B3 filaments (strong and non-diffusive staining) in the conditions indicated. Each color of square data points are from a separate biological replicate, the average for each biological replicate is displayed as an outlined circle in the corresponding color. The p value shown is from a paired two-tailed T-test comparing the biological replicate-level means (t = 16, two degrees of freedom). **(C)** Single-focal plane Airyscan images of expansion microscopy samples immunostained for lamin-B3 (red) and pore complexes (cyan, labeled by Mab414) in cleared egg extracts supplemented as indicated. Arrows in the Buffer/PEG 3350 image again highlight lamin-B3 signal that could lamin-B3 oligomers or short filaments, while arrowheads in the Ran-L43E/PEG 3350 image point to examples of filaments. Scale bars correspond to 1 μm pre-expansion, with an expansion factor of 3.9.

Transmission electron microscopy of membrane pellets prepared from our cleared egg extracts under control and lamin-B3 assembly conditions revealed annulate lamellae as electron-dense membrane stacks (Fig. S2A). Annulate lamellae were observed at a similar density in both conditions (Fig. S2B), indicating that Ran-L43E and PEG 3350 did not cause annulate lamellae proliferation in our experiments. Two-color STED imaging of egg extract samples treated with control and lamin-B3 assembly conditions revealed that lamin-B3 filaments decorate structures that contain NPCs (Fig. 3A) and membranes (Fig. S2C). Importantly, lamin-B3 assembly conditions caused significant enrichment of lamin-B3 signal on annulate lamellae (Fig. S2D), and visual inspection revealed that lamin-B3 filaments decorate 87±6.2% of all annulate lamellae observed (Fig. 3B). Although punctate lamin-B3 immunofluorescence signal was occasionally observed on annulate lamellae membranes under control conditions (Fig. 3A), filaments resolvable by STED imaging were rarely observed (Fig. 3A–B).

To better resolve NPCs and lamin filaments on the annulate lamellae, we applied expansion microscopy, which physically expanded samples around 4 times to enable imaging at a resolution of 30 nm when combined with Airyscan microscopy (44–46). Annulate lamellae in our samples strikingly resemble those observed by pan-expansion microscopy in HeLa cells and induced pluripotent stem cells (47), with individual NPCs resolved. As in our STED imaging experiments (Fig. 3A), treating extracts with control conditions (buffer and PEG 3350) resulted in residual, often punctate lamin-B3 signal on annulate lamellae. When extracts were treated with Ran-L43E and PEG 3350, annulate lamellae were decorated with numerous serpentine lamin-B3 filaments on the order of 1 μm in length (Fig. 3C), confirming our assembly conditions cause lamin-B3 to assemble on annulate lamellae.

Our data indicate that biochemical (i.e., presence of Ran-GTP) and biophysical (i.e., elevated colloid osmotic pressure) conditions that drive lamin-B3 assembly require an NPC-containing membrane that mimics the inner surface of the nuclear envelope, but not chromatin, nuclear transport, and other nuclear features. Therefore, the assembly of lamin filaments, a key component of the nucleus, can be separated from the rest of nuclear assembly.

To further interrogate the role of NPC-containing membranes in supporting lamin assembly, we asked whether the outside surface of nuclei could also support lamin-B3 assembly if we increased the colloid osmotic pressure and Ran-GTP concentration outside of assembled nuclei in the egg extracts. Indeed, ectopically elevating the cytoplasmic Ran-GTP concentration has previously been found to create nucleoplasmic structural and functional features in cytoplasm (28, 48).

**Figure 4:**
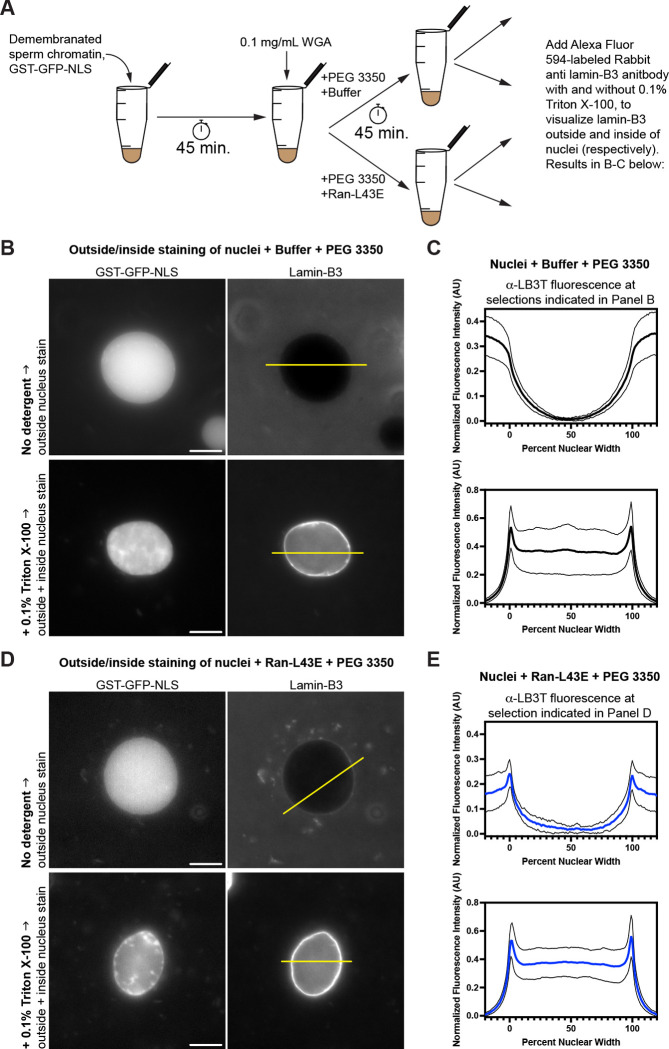
Lamin-B3 assembly can occur on the outside surface of the nuclear envelope **(A)** Schematic of the experiment used to determine whether lamin-B3 signal comes from the outside or inside surface of the nuclear envelope. Nuclei are stained with a rabbit anti lamin-B3 antibody decorated with Alexa Fluor 594-labled goat anti rabbit fab fragments, either in the absence or presence of 0.1% triton X-100. **(B)** Epifluorescence images of nuclei treated with control conditions (buffer and 1% PEG 3350) and stained as illustrated in panel A. GST-GFP-NLS signal delineates nuclei. Note that nuclei stained without triton X-100 were mounted without fixation, as fixation was found to compromise nuclear integrity, but those stained in the presence of triton X-100 were fixed with formaldehyde because they otherwise become misshapen upon mounting. Fixation accounts for the non-uniform GST-GFP-NLS signal. The yellow lines are examples of selections used for intensity profile analysis like that in panel C. Scale bars: 10 μm. **(C)** Mean (bold lines) ± SD (fine lines) intensity profiles for lamin-B3 for the corresponding conditions in panel B. Individual intensity profiles generated from selections like those shown in panel B were normalized to their maximum value, then the minimum value was uniformly subtracted to set the baseline to zero. Mean ± SD data are from measurements on 23 (top) and 22 (bottom) nuclei collected over the course of three independent experiments. **(D)** Epifluorescence images of nuclei prepared as in panel B, but treated with lamin-B3 assembly conditions (25 μm Ran-L43E and 1% PEG 3350). Scale bars: 10 μm. **(E)** Mean (bold lines) ± SD (fine lines) intensity profiles for lamin-B3 for the corresponding conditions in panel D. Mean ± SD data are from measurements on 18 (top) and 26 (bottom) nuclei collected over the course of three independent experiments. Data were treated as those in panel C.

We reconstituted nucleus assembly around demembranated sperm chromatin (21) in our cleared extracts in the presence of GST-GFP-NLS to label intact nuclei, then blocked further nucleocytoplasmic transport with 0.1 mg/mL wheat germ agglutinin (WGA) (49). When nuclei are assembled in *Xenopus* egg extracts, annulate lamellae formation is greatly reduced (41), so under these conditions, the major source of NPC-containing membranes present is the nuclear envelope itself. To the pre-assembled nuclei, we added fresh cleared extract, 1% PEG 3350, and either buffer or 25 μM Ran-L43E, then incubated the samples for a further 45 minutes. Nuclei were immediately labeled with our anti lamin-B3 antibody bound to an Alexa fluor 594-labeled Fab secondary antibody, which together functions as a fluorescent probe for lamin-B3 (50). Lamin-B3 labeling was performed either in the absence of detergent to limit access of the lamin-B3 probe to the outside of the reconstituted nuclei, or in the presence of 0.1% triton X-100 to allow labeling on both sides of the nuclei (Fig. 4A). Assembling the nuclei in the presence of GST-GFP-NLS allowed us to definitively identify intact nuclei in samples stained without detergent, such that the lamin-B3 probe would only have access to the outer nuclear surface.

For assembled nuclei treated with control conditions (buffer and PEG 3350), our experiment demonstrated that lamin-B3 was only present on the inside surface of the nuclear envelope (Fig. 4B–C, top panels) because the fluorescent lamin-B3 antibody probe was excluded from the nucleoplasm and did not decorate the nuclear boundary in the absence of detergent (Fig. 4B–C). By contrast, in the presence of 0.1% triton X-100, nuclei stained brightly with the lamin-B3 antibody probe (Fig. 4B–C, bottom panels). Therefore, lamin-B3 is only assembled on the inner surface of the nuclear envelope under this condition.

Strikingly, treating nuclei with lamin-B3 assembly conditions (Ran-L43E and PEG 3350), caused lamin-B3 to assemble on their outside surface, as our fluorescent lamin-B3 probe clearly labeled the boundary of these nuclei even without detergent present (compare Fig. 4D–E top panels to Fig. 4B–C top panels). As expected, labeling in the presence of detergent caused a strong labeling of the nuclear rim (Fig. 4D–E, bottom panels).

Here, we have reported conditions sufficient to reconstitute lamin-B3 assembly *in vitro* in a way that recapitulates many features of physiological lamin assembly. Our results demonstrate that elevated osmotic pressure and Ran-GTP drive lamin-B3 assembly on NPC-containing membranes, but they also reveal flexibility of the assembly pathway in that it can occur on a range of NPC-containing membranes including annulate lamellae and both the inner and outer surfaces of the nuclear envelope.

It has been speculated that purified lamins assemble filaments that self-associate laterally into paracrystals because of the absence of nuclear envelope transmembrane proteins in the “minimal” *in vitro* assembly system (18, 51). Our results suggest that the specific nuclear envelope proteins required for membranes to support lamin assembly are nucleoporins. We did not find well-known nuclear lamina proteins associated with the lamin-B3 filaments assembled in our egg extracts (Table S1), suggesting that such proteins are neither necessary for assembly, nor necessarily recruited upon assembly. Biochemical interactions between lamins and nucleoporins have been repeatedly reported (52, 53) and the distribution of lamin filaments and NPCs in the nucleus has been shown to be interdependent (40). While further studies are needed to clarify the specific roles of nucleoporins, especially those facing the nucleoplasm, in templating or organizing lamin assembly, our findings have set the stage to further dissecting the mechanism of lamin assembly both biochemically and structurally in normal and diseased states (54).

## Materials and Methods

### Plasmids, proteins, antibodies, and reagents

Recombinant GST-Ran-L43E was expressed from Zheng lab plasmid p213 in BL21(DE3) *E. coli* (New England Biolabs) and purified using standard approaches as previously reported (55–58). CyclinB-CDK1 was purchased from Millipore Sigma. Lamin-B3 was visualized in blots and by immunofluorescence using a lab-generated rabbit polyclonal antibody, generation of which was described previously (59). a-tubulin was visualized in blots using the DM1A mouse monoclonal antibody from Millipore Sigma. NPCs were visualized by immunofluorescence using the mouse monoclonal antibody Mab414 from Abcam, which recognizes several nucleoporins. Secondary antibodies used for immunoblots were the IR800 goat anti-rabbit antibody and the IR680 donkey anti-mouse antibodies from Licor. Secondary antibodies for immunofluorescence were the AlexaFluor 594 goat anti-rabbit antibody from Thermo Fisher and the ATTO647N goat anti-mouse antibody from Rockland. DiO and Hoechst were purchased from Thermo Fisher.

### Preparation of crude *Xenopus laevis* egg extracts

Egg extracts were generated using variations of three published protocols (21, 30, 60). Mature *X. laevis* females were injected with 50 units of pregnant mare serum gonadotropin (BioVendor) on day 1 and 25 units on day 3. To induce ovulation between days 5 and 12, the frogs were injected with 500 units of human chorionic gonadotropin (MP Biomedicals) and placed in 2 L of 1x MMR (5 mM HEPES, 100 mM NaCl, 2 mM KCl, 2 mM CaCl_2_, 1 mM MgCl_2_, 0.1 mM EDTA, pH 7.8). The next day, eggs were collected and rinsed 4–5 times with 1x MMR. Jelly coats were removed by treating for 5 minutes with 2% w/v cysteine made up in 100 mM KCl, 0.1 mM CaCl_2_, 1 mM MgCl_2_, pH adjusted to 7.8 with NaOH. Dejellied eggs were quickly rinsed 4–5 times with 0.2x MMR, then then activated by treating with 0.5 μg/mL calcium ionophore A23187 (Sigma-Aldrich) for 2 minutes. The eggs were then washed 5 times with extract buffer (“XB:” 100 mM KCl, 0.1 mM CaCl_2_, 1 mM MgCl_2_, 10 mM HEPES, 50 mM Sucrose, pH 7.7), twice with XB+ (XB with 10 μg/mL leupeptin, pepstatin, and chymostatin), and transferred to ultra-clear 13 × 51 mm ultracentrifuge tubes (Beckman). 15 minutes after adding the ionophore, the eggs were packed in a Damon/IEC Division HN-SII centrifuge by setting the speed control to “full,” spinning until the speed reached 1500 rpm, then immediately turning the instrument off and allowing the rotor to stop on its own. The packed eggs were placed on ice for 15 minutes, then crushed by centrifuging for 15 minutes at 10,000 rpm (~12,000 ×g) and 4°C in an SW 55 Ti rotor (Beckman). The layer composed of golden-colored cytoplasm was slowly withdrawn using a 1 mL syringe with an 18-gauge needle, then expelled into 1.5 mL microcentrifuge tubes on ice and supplemented with 10 μg/mL leupeptin, peptstatin, and chymostatin, 133 μg/mL cycloheximide, and energy mix (4 mM creatine phosphate, 0.4 mM ATP, 20 μg/mL creatine kinase, 0.4 mM MgCl_2_).

### Preparation of cleared egg extracts

2.2 mL of fresh crude extract prepared as described above was transferred into an ultra-clear 11 × 34 mm ultracentrifuge tube (Beckman) and centrifuged for 45 minutes at 50,000 rpm (~166,000 ×g) and 4°C in a TLS-55 rotor (Beckman). Following centrifugation, the extract is stratified into several major layers. From the top, they are: lipids, cytosol, light membranes, cytosol with some membranes mixed in, heavier membranes, and glycogen (30). Using a wide bore pipet, both cytosolic layers and the light membrane layer were collected and mixed. This “cleared extract” was snap frozen in 26 μL aliquots and stored in liquid nitrogen.

### Reconstitution of nuclear assembly and lamin-B3 assembly in egg extracts

Reconstitution of nuclear assembly was achieved by adding 150,000–200,000 demembranated sperm chromatin masses (21) directly to 24 μL crude or cleared egg extract. Nuclear assembly was monitored over time by mixing 2 μL of the reaction with an equal volume of nucleus fix (25 mM HEPES pH 7.7, 500 mM sucrose, 8% formaldehyde, 10 μg/mL Hoechst 33350). > 90% of the chromatin masses routinely assembled into nuclei within 40 minutes.

To trigger lamin-B3 assembly, extracts were supplemented with 25 μM Ran-L43E alone (crude extracts) or with 25 μM Ran-L43E and 1% w/v PEG 3350 (cleared extracts). A nuclear assembly reaction was routinely run in parallel as a positive control for extract quality and following the reasoning that the time course of lamin-B3 assembly would be at least as fast as nuclear assembly.

### Immunofluorescence sample preparation

We found that lamin-B3 structures adhered to both normal glass coverslips and the poly-L-lysine coated coverslips. Lamin-B3 assembly reactions were pipetted onto clean #1.5H cover glasses, then an equal volume of 2x XB with 5% formaldehyde was added. Samples were fixed for 10 minutes, then quenched by adding glycine to a final concentration of 125 mM and incubating for 5 minutes. The liquid was aspirated and replaced with 3% w/v bovine serum albumin (BSA) made up in phosphate-buffered saline (PBS). After blocking for 30 minutes, samples were stained with the indicated primary antibodies at 3–6 μg/mL in PBS with 3% BSA for 30 minutes, then washed with five immediate exchanges of PBS with 0.1% v/v Igepal CA-630 (Sigma-Aldrich). For epifluorescence or STED imaging, samples were stained with secondary antibodies at 1.3 μg/mL in PBS with 3% BSA, washed with 5 immediate exchanges of PBS with 0.1% v/v Igepal CA-630, and mounted in Prolong Diamond (ThermoFisher).

### Expansion microscopy sample preparation

Samples were prepared as for immunofluorescence, but after primary antibody staining, the samples were incubated for 1 hour with goat anti rabbit antibody (Jackson Immunoresearch, Cat#111-005-144) custom conjugated with AlexaFluor488 (Lumiprobe, Cat#21820) and goat anti mouse antibody (Jackson Immunoresearch, Cat#115-005-146) custom conjugated with AlexaFluor 568 (Invitrogen, Cat#A20003), each at a concentration of 6 μg/mL in PBS with 0.1% triton X-100 (61). After two five-minute PBS washes, the samples were incubated in 100 mM sodium bicarbonate for five minutes, then incubated in 0.04% (w/v) glycidyl methacrylate (GMA) (Sigma, Cat#151238) in 100 mM sodium bicarbonate for three hours at room temperature. The GMA-modified sample was washed with PBS for five minutes three times, then incubated with monomer solution (8.6 g sodium acrylate, 2.5 g acrylamide, 0.15 g N,N’-methylenebisacrylamide, 11.7 g sodium chloride in 94 ml PBS) on ice for 5 min. Gelation solution (mixture of monomer solution, 10% (w/v) N,N,N,N Tetramethylethylenediamine (TEMED) stock solution, 10% (w/v) ammonium persulfate (APS) stock solution and water at 47:1:1:1 volume ratio) was then added and samples were incubated for another five minutes on ice. The sample was then gelated at 37°C in a humidity chamber for 1 hour. Following gelation, the sample was gently detached from the cover slip with a forceps in heat denaturation buffer (200 mM sodium dodecyl sulfate, 200 mM NaCl, and 50 mM Tris pH 6.8), then immersed in heat denaturation buffer for 1.5 hours at 78°C. After heat denaturation, the gelated sample was fully immersed in excess DNase/RNase-free water for 30 minutes three times before being expanded in water overnight. The fully expanded gelated sample (~3.9 times expansion) was trimmed and transferred to a glass bottom dish for imaging.

### Fluorescence microscopy

STED imaging was carried out on a Leica TCS SP8 STED 3X imaging system with HyD detectors on a Leica DMi8 stand with either an HC PL APO 86x/1.2 NA W motCORR objective or an HC PL APO 100x/1.4 NA OIL CS2 objective. The excitation wavelength for AlexaFluor 592 was 590 nm, with detection in the range of 609–684 nm (single channel images) or 604–641 nm (dual color images). Atto 647N was excited at 653 nm and detected from 665–740 nm. For both fluorophores, a 775 nm depletion laser was used for STED imaging.

Expansion microscopy samples were imaged on a ZEISS LSM 980 with Airyscan 2 using a 63x water-immersion objective (Zeiss LD C-Apochromat 63x/1.15 W Corr M27). Airyscan SR and best signal mode with 0.2 AU pinhole and 1.25 AU total detection area was used for all expanded samples. The effective lateral resolution of Airyscan microscope was measured by TetraSpeck^™^ Microspheres (0.1 μm, fluorescent blue/green/orange/dark red) at 138 nm. After combining with expansion microscopy, the actual lateral resolution was enhanced to ~35 nm.

Epifluorescence imaging was carried out on a Nikon Eclipse E800 microscope with a Nikon Plan Apo 60x/1.4 NA oil immersion objective, a Hamamatsu ORCA-Flash 4.0 LT+ sCMOS camera, and an Excelitas X-Cite 120LEDmini LED illumination system. Filter sets used were Semrock’s GFP filter set (excitation filter FF01–466/40–25, emission filter FF03–525/50–25) and Texas Red filter set (excitation filter FF01–562/40–25, emission filter FF01-62440-25).

### Sucrose gradient sedimentation experiments

5–50% sucrose gradients (1.25 mL total) were poured as step gradients (equal volumes of 50%, 38.75%, 27.5%, 16.25%, and 5% sucrose) in XB and allowed to diffuse into continuous gradients overnight in 11 × 34 mm polycarbonate ultracentrifuge tubes. Extract reactions were diluted 1:1 with XB and overlayed onto the gradients, then centrifuged for 4.5 hours at 50,000 rpm (~166,000 ×g) at 4°C in a TLS-55 rotor. After sedimentation, the gradients were fractionated from the top using wide-bore pipet tips. Samples from each fraction were mixed with an equal volume of 2x tris urea sample buffer (125 mM Tris pH 6.8, 6 M urea, 2% sodium dodecyl sulfate, 10% 2-mercaptoethanol), boiled for 5 minutes, and resolved on 12% polyacrylamide gels. Sedimentation patterns were visualized by immunoblotting following manufacturer-recommended procedures (Bio-Rad) and imaging on a Licor Odessey CLx infrared scanner. Band intensities were measured using the associated ImageStudio software.

### Proteomics

3 biological replicate cleared egg extract samples treated with lamin-B3 assembly conditions (25 μM Ran-L43E and 1% PEG 3350) or with control conditions (buffer only) were subjected to sucrose gradient sedimentation as described above, and the tenth fraction was processed for proteomic analysis. Samples were run < 1 cm into a precast 4–12% polyacrylamide gel (Bio-Rad) and the gel was stained with Coomassie Brilliant Blue G-250 using standard procedures to reveal the boundaries of each lane. The lanes were individually excised using a clean razor blade.

In-gel trypsin digestion was performed by washing each gel after dicing it with a clean razor blade. Gel samples were repeatedly washed with 25 mM ammonium bicarbonate/50% ACN. Afterward, the samples were reduced with 10 mM DTT, alkylated with 50 mM IAM, and dried using a speed vac. Trypsin was then added and the samples were digested overnight at 37°C. The peptides were extracted by vortexing with 50% ACN/0.1% formic acid, then desalted using C18 ZipTips (Millipore).

LC-MS/MS was carried out on a Orbitrap Eclipse Tribrid Mass Spectrometer (Thermo Fisher), equipped with an Easy LC 1200 UPLC liquid chromatography system (Thermo Fisher). Peptides were first trapped using a trapping column (Acclaim PepMap 100 C18 HPLC, 75 μm particle size, 2 cm bed length), then separated using analytical column AUR3-25075C18, 25CM Aurora Series Emitter Column (25 cm × 75 μm, 1.7 μm C18) (IonOpticks). The flow rate was 300 nL/min. Peptides were eluted by a gradient from 3 to 28 % solvent B (80 % acetonitrile, 0.1 % formic acid) over 106 min and from 28 to 44 % solvent B over 15 min, followed by a short wash (15 min) at 90 % solvent B.

Data dependent acquisition was carried out with the following parameters. Precursor scan was from mass-to-charge ratio (m/z) 375 to 1600 (resolution 120,000; AGC 200,000, maximum injection time 50ms, Normalized AGC target 50%, RF lens(%) 30) and the most intense multiply charged precursors were selected for fragmentation (resolution 15,000, AGC 5E4, maximum injection time 22ms, isolation window 1.4 m/z, normalized AGC target 100%, include charge state=2–8, cycle time 3 s). Peptides were fragmented with higher-energy collision dissociation (HCD) with normalized collision energy (NCE) 27. Dynamic exclusion was enabled for 30s.

Peptide identification and quantification of the mass spectrometry data was carried out using MaxQuant Version 2.4.10.0 (62). All three control and experimental samples were analyzed simultaneously using the default settings with matching between runs turned on. Label free quantification (LFQ) was enabled with the minimum ratio count set to two. The mass spectrometry data were searched against a database of 34,809 protein sequences (Proteome ID: UP000186698, available at download.xenbase.org/xenbase/Proteomes/, RRID:SCR_003280) generated from the *Xenopus laevis* v10.1 genome assembly (NCBI RefSeq: GCF_017654675.1). A reverted database was used to determine the false discovery rate and set it to 1%.

To analyze the resulting proteomic data, the identified protein groups and their LFQ intensities from the MaxQuant search were loaded into Perseus Version 2.0.11.0 (63). Data were filtered to remove decoys, potential contaminants, and protein groups that were only identified by site (i.e., posttranslational modification). LFQ intensities were log_2_ transformed, and only protein groups that had valid values for either all three experimental samples or all three control samples were kept, an operation that eliminated non-reproducible identifications. Remaining invalid values were replaced with imputed values from a normal distribution downshifted by 1.8 with a width of 0.3 made from all matrix values. A two-sided T-test with S_0_ = 1 and FDR = 0.05 was used to determine the significance and enrichment of each protein group. The volcano plot as displayed in Fig. 2A was generated in Graphpad Prism Version 10.5.0.

### Transmission electron microscopy sample preparation and imaging

Electron microscopy sample preparation and image acquisition was carried out essentially as described in (41). Cleared extract samples treated with either control conditions (buffer and 1% PEG 3350) or lamin-B3 assembly conditions (25 μM Ran-L43E and PEG 3350) were incubated for 40–60 minutes at 25°C, then fixed in 112.5 mM cacodylate (pH 7.4) and 3% formaldehyde for 5 minutes at room temperature and 25 minutes at 4°C. Fixed samples were then centrifuged for 10 minutes at 3000 ×g at room temperature to pellet membranes. The pellet was washed once with 0.1 M cacodylate buffer (pH 7.4) then enrobed in 2% low melt agarose made up in 0.1 M cacodylate buffer. Agarose-enrobed pellets were washed with 0.1 M cacodylate with 50 mM glycine to quench any residual formaldehyde, then rinsed with 0.1 M cacodylate buffer and stained with 2% osmium tetroxide in 0.1 M cacodylate buffer. Osmium tetroxide-stained samples were rinsed thoroughly with water, then stained for 15 minutes with 1% uranyl acetate. Samples were again rinsed thoroughly with water, then dehydrated through a concentration series into anhydrous ethanol and embedded in araldite.

Electron microscopy images were acquired on a Hitachi HT7800 transmission electron microscope operated at 80 KeV at 2000x magnification using an AMT Nanosprint 12 camera.

## Supplementary Material

Supplement 1

Supplement 2

## Data Availability

All data needed to evaluate the conclusions in the paper are present in the paper and/or the Supplementary Materials.
