# Supplementary material for: Reconstitution of lamin assembly on nuclear pore complex-containing membranes
Source: bioRxiv. 2025 Jul 30:2025.07.28.667287. Preprint. [Version 1] doi: 10.1101/2025.07.28.667287 (PMC12324298; doi:10.1101/2025.07.28.667287)

bioRxiv preprint doi: https://doi.org/10.1101/2025.07.28.667287; this version posted July 30, 2025. The copyright holder for this preprint (which was not certified by peer review) is the author/funder, who has granted bioRxiv a license to display the preprint in perpetuity. It is made available under aCC-BY 4.0 International license.

Figure S1: Lamin-B3 assembly can be reconstituted in crude *Xenopus* egg extracts, but they are not suitable for stimulated emission depletion (STED) imaging

(A) Epifluorescence images of crude *Xenopus* egg extracts treated with a buffer control or with ~25 μM Ran-L43E and stained by lamin-B3 immunofluorescence. Ran-L43E, but not crowding agent, is needed for lamin-B3 assembly in crude extracts, possibly because abundant glycogen present in crude extracts serves a similar role. Scale bar: 5 μm.

(B) Illustration of one reason why crude extracts are not suitable for systematic studies of lamin-B3 assembly. Lamin-B3 assembly was triggered by adding Ran-L43E to crude extracts, as in panel A. Left: conventional confocal and DIC image of lamin-B3 structures before attempted STED imaging. DIC imaging reveals many small shiny granules present in the sample (arrowheads). Center: Attempted STED fluorescence image. The granules are excited by the depletion laser and saturate the detectors, masking lower intensity signals. Right: conventional confocal and DIC image of lamin-B3 structures after attempted STED

bioRxiv preprint doi: https://doi.org/10.1101/2025.07.28.667287; this version posted July 30, 2025. The copyright holder for this preprint (which was not certified by peer review) is the author/funder, who has granted bioRxiv a license to display the preprint in perpetuity. It is made available under aCC-BY 4.0 International license.

imaging. The small granules explode, physically distorting the sample (arrowheads). Scale bar: 5 µm.

Figure S2: Conditions that promote lamin-B3 assembly do not alter membranes or cause annulate lamellae proliferation in cleared *Xenopus* egg extracts

(A) Transmission electron micrographs of membrane pellets prepared from cleared *Xenopus* egg extracts treated as indicated, stained with osmium tetroxide, resin-embedded, ultrathin-sectioned, and counterstained with uranyl acetate. One example annulate lamellae membrane stack is indicated in a yellow box in each image. Note that some annulate lamellae are captured in the section in profile such that the membrane stack is clearly visible (yellow arrow), while others are captured *en face* (yellow arrowhead) such that the dense pore complexes within are apparent. Scale bars: 5 µm with 500 nm ticks. **(B)** Quantification of the density of annulate lamellae observed in images like those shown in panel A. Each color of circle data points is from a separate biological replicate, the average for each biological replicate is displayed as an outlined triangle in the corresponding color. The p value shown is from a paired two-tailed T-test comparing the biological replicate-level means ($t = 0.3674$, one degree of freedom). **(C)** Maximum intensity-projected two-color immunofluorescence images of lamin-B3 (red, imaged by STED) and membranes (cyan, labeled by DiO, imaged by conventional confocal imaging), in cleared egg extracts supplemented as indicated. Scale bars: 5 µm. **(D)** A second quantification of the experiment from Fig. 3A: total lamin-B3 signal per annulate lamellae area, measured in two separate images from three biological replicates. The p value shown is from a paired two-tailed T-test comparing the biological replicate-level means ($t = 8.654$, two degrees of freedom).

bioRxiv preprint doi: https://doi.org/10.1101/2025.07.28.667287; this version posted July 30, 2025. The copyright holder for this preprint (which was not certified by peer review) is the author/funder, who has granted bioRxiv a license to display the preprint in perpetuity. It is made available under aCC-BY 4.0 International license.

Supplemental Figure S1

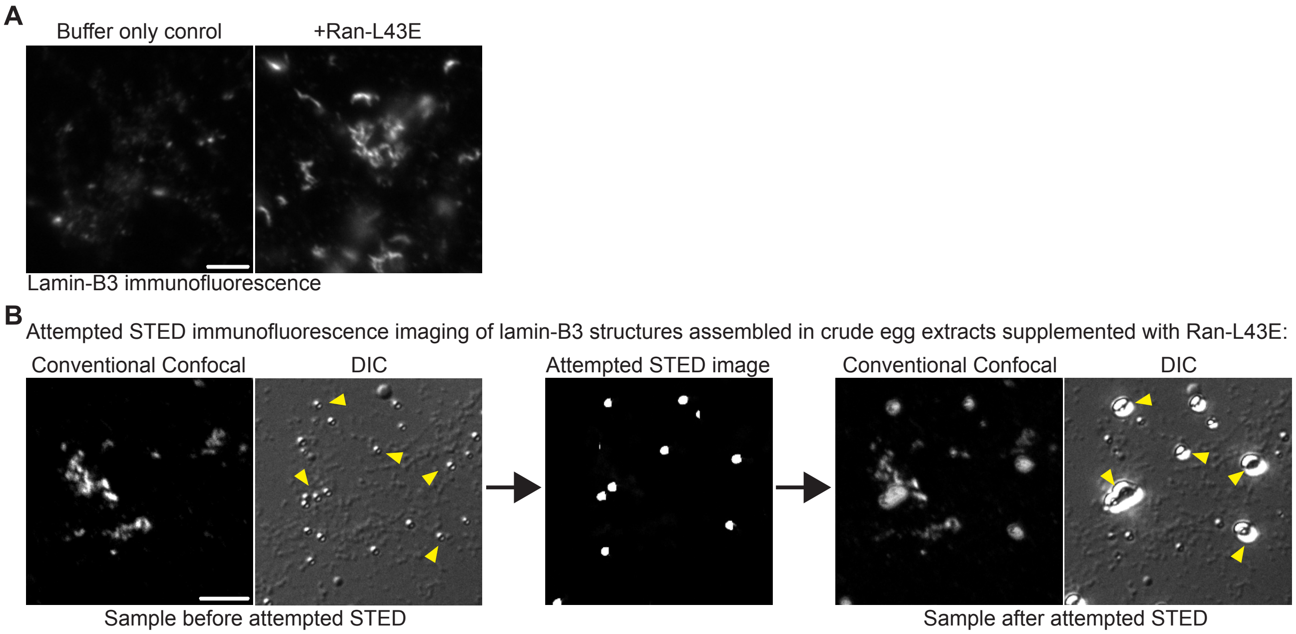

bioRxiv preprint doi: https://doi.org/10.1101/2025.07.28.667287; this version posted July 30, 2025. The copyright holder for this preprint (which was not certified by peer review) is the author/funder, who has granted bioRxiv a license to display the preprint in perpetuity. It is made available under aCC-BY 4.0 International license.

Figure S2

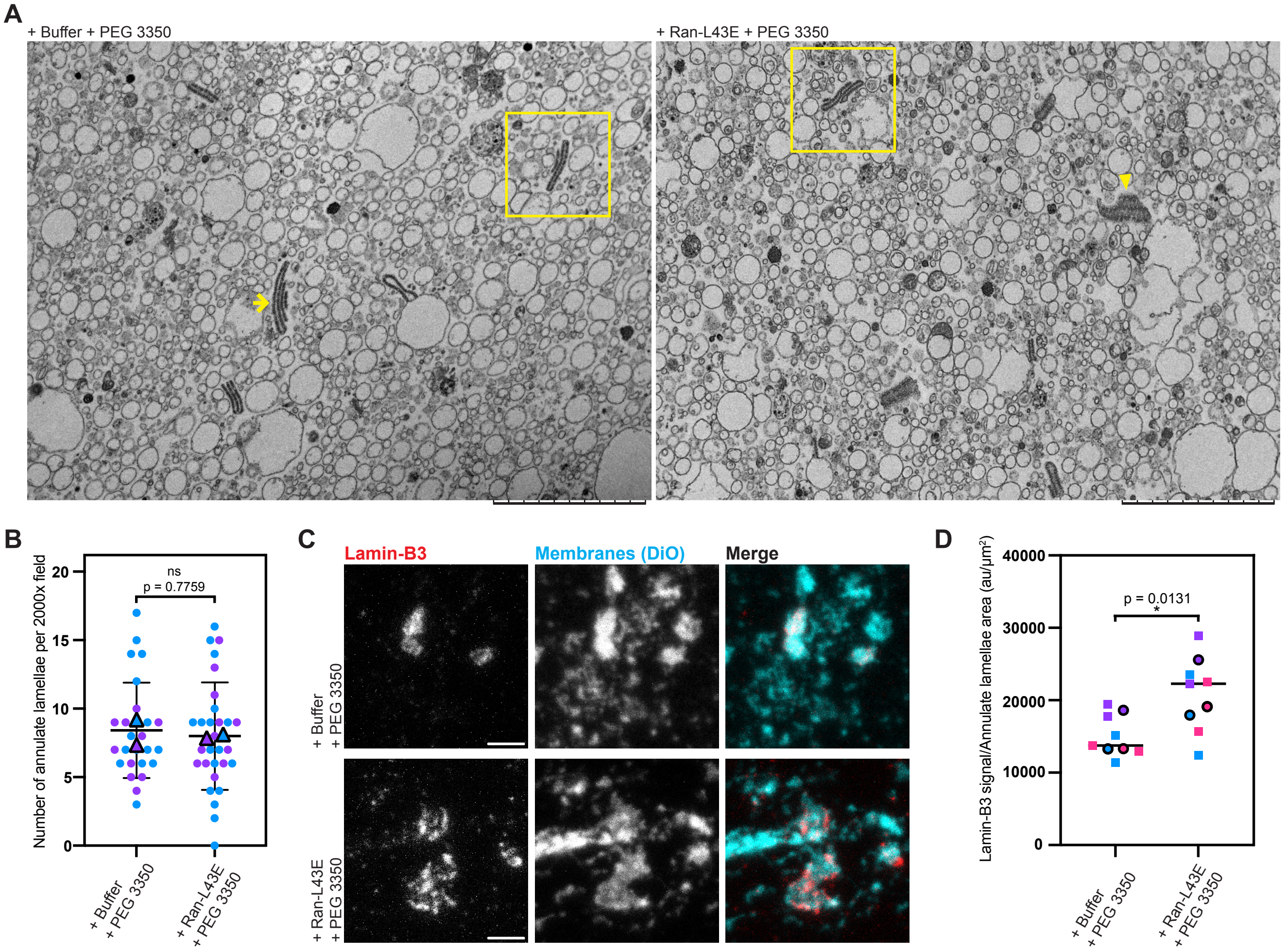

Supplement: Supplement 2 [file NIHPP2025.07.28.667287v1-supplement-2.pdf]
